# Population Decline May Affect the Incidence of Pulmonary Tuberculosis in Japan

**DOI:** 10.3390/jcm15176745

**Published:** 2026-08-30

**Authors:** Akihiro Miyauchi, Mizuki Inaba, Takatoshi Tetsuka, Noritaka Sakamoto, Yushi Nomura, Otohiro Katsube, Hirokuni Hirata, Hiroshige Yoshioka, Seiji Niho, Kumiya Sugiyama

**Affiliations:** 1 Department of Respiratory Medicine and Clinical Immunology, National Hospital Organization Utsunomiya Hospital, Tochigi 329-1193, Japan; a-miyauchi@dokkyomed.ac.jp (A.M.); m-toyoda781@dokkyomed.ac.jp (M.I.); t-tetsuka@dokkyomed.ac.jp (T.T.); nori.smoto@gmail.com (N.S.); uc.nomura@gmail.com (Y.N.); 5okatsube@gmail.com (O.K.); 2 Department of Respiratory Medicine and Clinical Immunology, Dokkyo Medical University Saitama Medical Center, Saitama 343-8555, Japan; hirokuni@dokkyomed.ac.jp (H.H.); h-yoshioka180@dokkyomed.ac.jp (H.Y.); 3 Department of Pulmonary Medicine and Clinical Immunology, Dokkyo Medical University, Tochigi 321-0293, Japan; siniho@dokkyomed.ac.jp

**Keywords:** epidemiology, foreigner, Japan, population decline, tuberculosis

## Abstract

**Background/Objectives**: Pulmonary tuberculosis in Japan had been decreasing as a result of the country’s anti-tuberculosis policy. The decreased number of patients with tuberculosis has led to many tuberculosis units being downsized. However, the decreasing trend in incidence ended in 2022. To resume this trend, it is important to clarify which populations are at higher risk of developing tuberculosis. **Methods**: This study analyzed annual reports on tuberculosis published by the Japanese government from 2015 to 2024. **Results**: Among Japanese nationals, the number of newly notified cases of tuberculosis had been decreasing in all age groups. However, the number of newly notified cases among foreign-born residents has sharply increased since 2022, and the rate of newly notified cases among foreign-born residents per 100,000 population was highest in the 20–39-year age group. During the observation period, the number of foreign-born workers from countries with a high incidence of tuberculosis had increased, and the populations of several countries and the corresponding numbers of tuberculosis notifications increased. **Conclusions**: This study revealed that younger foreign workers from those countries are more likely to develop pulmonary tuberculosis in Japan. The change in population composition may have impacted the incidence of tuberculosis in Japan and will be a hindrance to efforts to decrease the number of tuberculosis cases going forward. Therefore, more effective pre-entry tuberculosis screening should be introduced for young migrant workers from countries with a high incidence of tuberculosis cases.

## 1. Introduction

Pulmonary tuberculosis is one of the most consequential infectious diseases globally because it carries a risk of death for older people and people with immunosuppression [1]. Under Japanese law, pulmonary tuberculosis must be treated in an isolated tuberculosis unit. The number of pulmonary tuberculosis cases in Japan decreased by nearly half, from 21,283 (16.7 cases per 100,000 population) in 2012 to 10,051 cases (8.1 cases per 100,000 population) in 2024 as a result of increased tuberculosis screening in offices and schools [2,3,4]. Although the anti-tuberculosis policy had proved effective, the decreasing trend ended in 2022, with incidences of 8.2 and 8.1 cases per 100,000 population in 2022 and 2024, respectively [2,3]. Meanwhile, the numbers of foreign-born cases increased from 1069 in 2012 to 1980 in 2024 [2,3]. As of 2024, Japan has more than 3.5 million resident aliens [5], with many technical interns and workers coming from countries with a high incidence of pulmonary tuberculosis [2,3,4,6,7]. Among these residents, the top 5 countries of origin with the most pulmonary tuberculosis cases in 2024 were Indonesia, the Philippines, Nepal, Myanmar, and Vietnam, with 423, 331, 293, 280, and 252 cases per year, respectively [3]. The influx of migrant workers from those countries may have contributed to the increasing incidence of pulmonary tuberculosis in Japan since 2022.

In Japan, the occupancy rate for tuberculosis units has decreased, leading to problems in hospital management because the number of patients with pulmonary tuberculosis had been decreasing for a long period of time. Accordingly, tuberculosis units have already been downsized in some prefectures. However, tuberculosis units should only be reorganized after appropriate analysis, given that tuberculosis units require isolated depressurized rooms and cannot be expanded in a short period of time. To ensure proper operation of tuberculosis units, it is important to predict the future incidence of pulmonary tuberculosis cases.

A study in the European Union suggested that migrants arriving from high-incidence tuberculosis countries may pose a threat to tuberculosis control in low incidence European host countries [8]. In Japan, migrants may also have contributed to the end of the decreasing trend in tuberculosis incidence. Although various annual reports on tuberculosis and the population composition have been published by different ministries or agencies, the precise reasons for the end of the decreasing trend have not been determined. Therefore, this study analyzed the relationship between population composition and the incidence of pulmonary tuberculosis in Japan.

## 2. Materials and Methods

### 2.1. Study Design

This retrospective study analyzed data from the annual report on pulmonary tuberculosis in Japan published by the Ministry of Health, Labour and Welfare. These reports include demographic data such as residential location, age, and nationality. The population composition in Japan has been published every year by the Statistics Bureau of Japan and includes the populations of Japanese nationals and foreign residents. Statistical data, including age group and residence status according to nationality, are published by the Immigration Service Agency. In this study, we analyzed data for the period of 2015–2024 and performed an ecological analysis of aggregate surveillance data.

In the analysis by age group, patients were divided into the following four age groups based on the above publication: 19 years and under, 20–39 years, 40–59 years, and 60 years and over. In the analysis of the newly notified cases of pulmonary tuberculosis per 100,000 population, the same population categories were used; for example, the population of foreign residents was used for the analysis of foreign-born patients, and the population of patients aged 20–39 years was used for the analysis of incidence in the 20–39-year age group. In the analysis of age-standardized rates, the population of Japanese nationals in 2024 was chosen as the reference standard population.

### 2.2. Data Analysis

Data were analyzed using Excel 2024 ver. 2604 (Microsoft Corporation, Redmond, WA, USA).

## 3. Results

### 3.1. Populations

**Figure 1:**
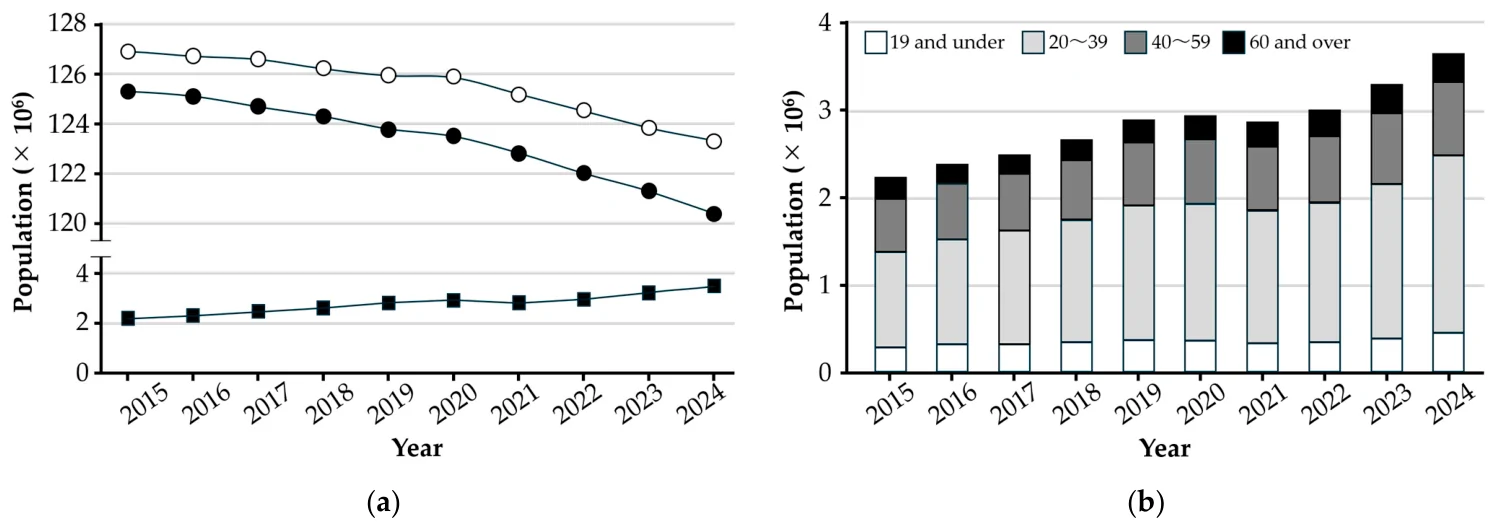
The total population of Japan (open circles), the population of Japanese nationals (closed circles), and the population of foreign residents (closed squares) are shown in (**a**). The population of foreign residents according to age group is shown in (**b**).

The total population of Japan, the population of Japanese nationals, and the population of foreign residents are shown in Figure 1a [5]. The Japanese population has decreased while the population of foreign residents has increased, except during the period of the COVID-19 pandemic. The population of foreign residents according to age group is shown in Figure 1b. Each age group, except the 20–39-year age group, grew slightly during the 10 years of the observation period, while the 20–39-year age group increased dramatically, from 1.1 million in 2015 to 1.5 million in 2019. Although these increasing trends were stopped by the restrictions on entry during the COVID-19 pandemic, the 20–39-year age group resumed its rapid growth after the pandemic, increasing from 1.6 million in 2022 to 2.0 million in 2024.

#### 3.1.1. Number of Newly Notified Cases of Pulmonary Tuberculosis

**Figure 2:**
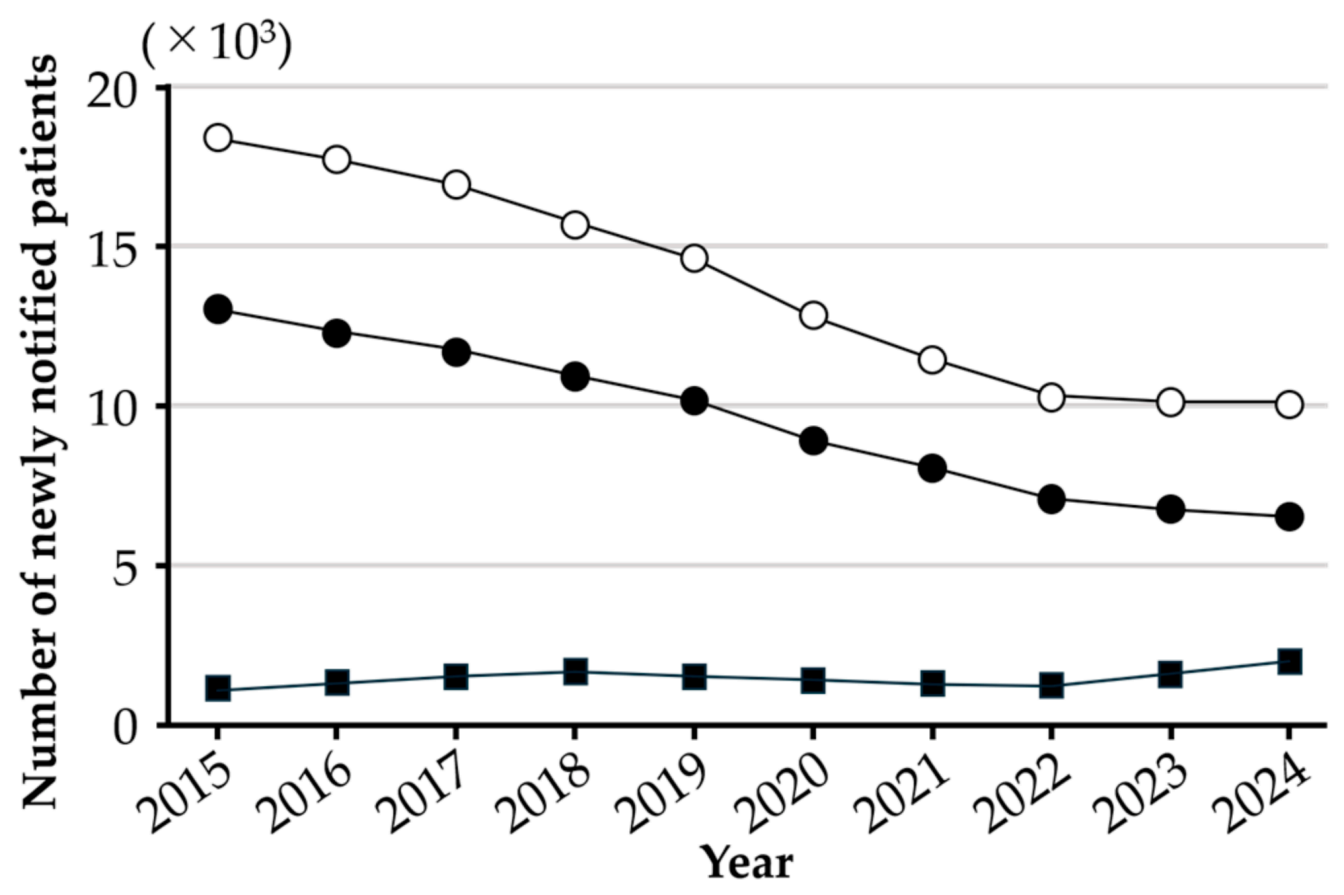
The total number of patients with pulmonary tuberculosis in Japan (open circles), the number of Japanese nationals with pulmonary tuberculosis (closed circles) and the number of foreign-born residents with pulmonary tuberculosis (closed squares) are shown.

The total number of newly notified cases of pulmonary tuberculosis in Japan and among Japanese nationals, and the number of foreign-born patients with pulmonary tuberculosis in Japan are shown in Figure 2. The decreasing trend in Japan ended in 2022, with 10,235, 10,096, and 10,051 cases in 2022, 2023, and 2024, respectively. The numbers of newly notified cases of pulmonary tuberculosis among Japanese nationals were 8673, 8206, and 7911 cases in 2022, 2023, and 2024, respectively. The number of newly notified cases of pulmonary tuberculosis among foreign-born residents in Japan increased from 1164 in 2015 to 1667 in 2018. The number decreased due to restrictions on traveling to Japan during the COVID-19 pandemic but then increased from 1214 in 2022 to 1980 in 2024.

#### 3.1.2. Rate of Newly Notified Cases of Pulmonary Tuberculosis

**Figure 3:**
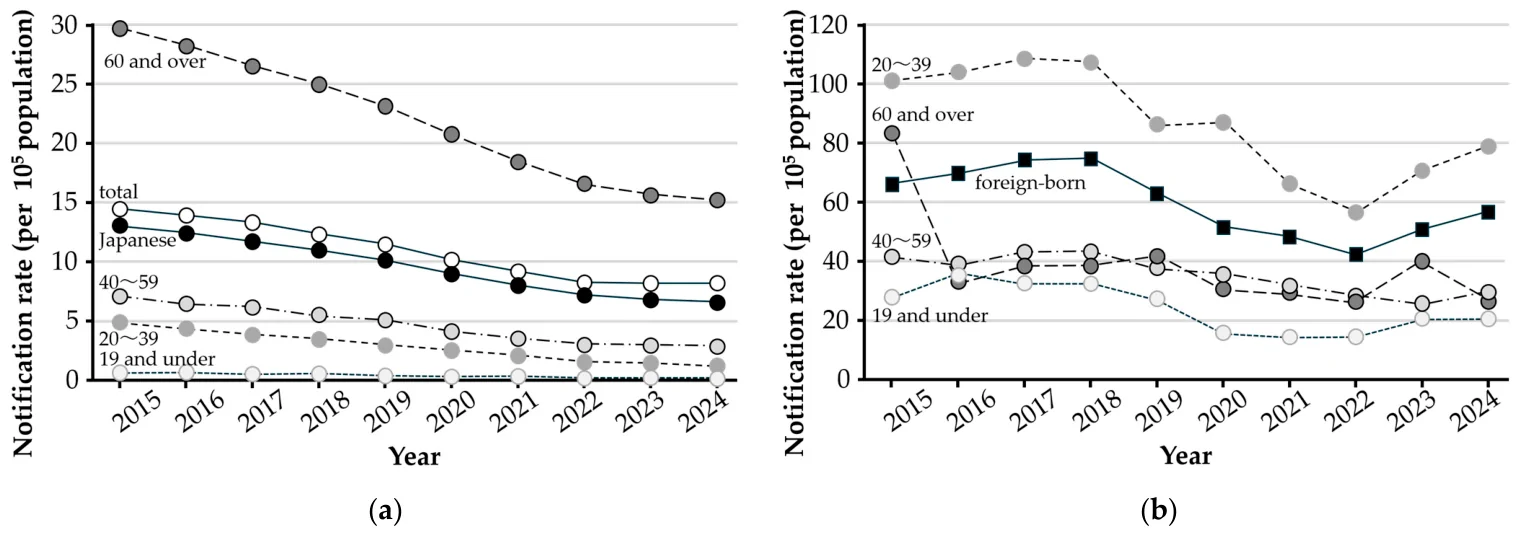
(**a**) Rates of newly notified cases of pulmonary tuberculosis per 100,000 population in Japan as a whole, among Japanese nationals, and among Japanese age groups. (**b**) Rates among foreign-born residents according to age groups.

The rates of newly notified cases of pulmonary tuberculosis per 100,000 population in Japan as a whole and among Japanese nationals are shown in Figure 3a, and that among foreign-born residents is shown in Figure 3b. Japan is a super-aged society, and the population composition of Japanese nationals differs from that of foreign residents. To compare the different population compositions, the notification rates by age group among Japanese nationals and foreign-born residents are shown in Figure 3a and b, respectively. In addition, the age-standardized rates of newly notified cases of pulmonary tuberculosis per 100,000 population among Japanese nationals and foreign-born residents in Japan are shown in Table 1.

As shown in Figure 3a, the decreasing trend in Japan ended in 2022, with an incidence of 8.2 and 8.1 cases per 100,000 population in 2022 and 2024, respectively. Among Japanese nationals, the decreasing trend also slowed after 2022. Higher rates were observed in older age groups, and the rates were decreasing in all age groups. As shown in Figure 3b, among foreign-born patients, the rates in the 20–39-year age group were higher than those in other age groups. Notification rates decreased because of the COVID-19 pandemic but then increased again. This suggests that foreign residents aged 20–39 years are more likely to developing pulmonary tuberculosis in Japan. As shown in Figure 1b, due to restrictions on traveling to Japan, the population of foreign residents did not increase during the COVID-19 pandemic. However, the number of foreign nationals entering has increased since the end of the COVID-19 pandemic, and the incidence rate in the high-risk group has increased again.

### 3.2. Number of Foreign-Born Residents with Pulmonary Tuberculosis and the Incidence Rates in Their Countries of Origin

The numbers of newly notified cases of pulmonary tuberculosis among foreign-born residents in Japan in 2024 according to country of origin are shown in Table 2 [3]. The rates of newly notified cases of pulmonary tuberculosis per 100,000 population in these countries in 2024 are also shown in Table 2 [7]. To evaluate the reasons for the change in incidence in 2022, the relationships between the number of foreign-born patients and the foreign population were analyzed. The top five countries in terms of incidence for pulmonary tuberculosis are analyzed below.

#### 3.2.1. Relationship Between the Number of Indonesian Patients with Pulmonary Tuberculosis and the Indonesian Population in Japan

**Figure 4:**
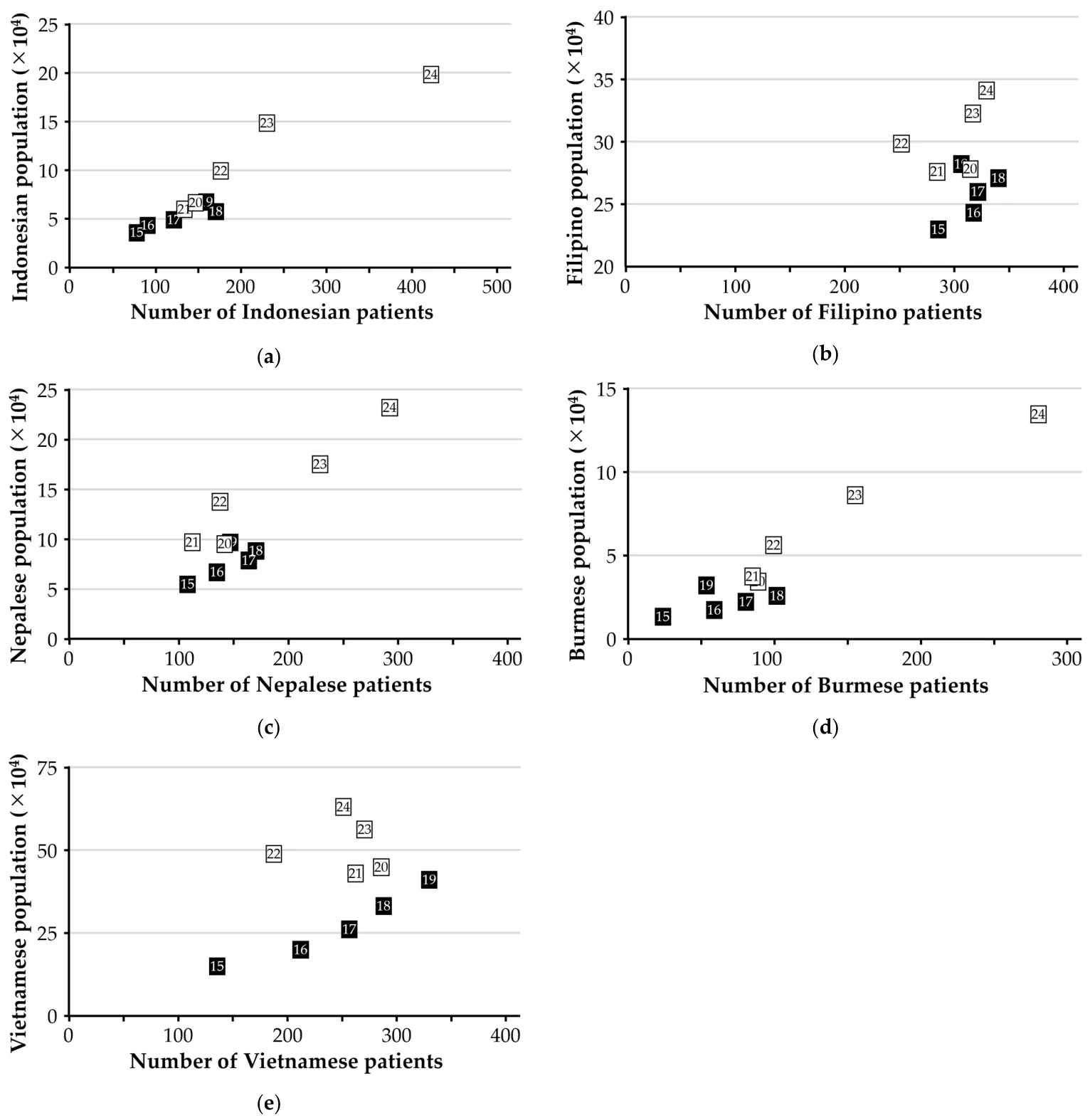
Relationship between foreign-born populations in Japan and the number of foreign-born residents with pulmonary tuberculosis in Japan, (**a**) Indonesian; (**b**) Filipino; (**c**) Nepalese; (**d**) Burmese; (**e**) Vietnamese. Closed squares show data from 2015 to 2019, and open squares show data from 2020 to 2024. Numbers in squares show the last 2 digits of the year.

The largest group of foreign-born residents with pulmonary tuberculosis in Japan in 2024 by country was from Indonesia. In 2024, Indonesia had 382 patients with pulmonary tuberculosis per 100,000 population. The number of Indonesian patients with pulmonary tuberculosis in Japan increased from 78 patients in 2015 to 423 patients in 2024. The relationship between the number of Indonesian residents with pulmonary tuberculosis and the Indonesian population in Japan is shown in Figure 4a.

#### 3.2.2. Relationship Between the Number of Filipino Patients with Pulmonary Tuberculosis and the Filipino Population in Japan

The second largest group of foreign-born residents with pulmonary tuberculosis in Japan in 2024 by country was from the Philippines. In 2024, the Philippines had 625 patients with pulmonary tuberculosis per 100,000 population, the highest incidence among the 10 countries analyzed. The relationship between the number of Filipino residents with pulmonary tuberculosis and the Filipino population in Japan is shown in Figure 4b.

#### 3.2.3. Relationship Between the Number of Nepalese Patients with Pulmonary Tuberculosis and the Nepalese Population in Japan

The third largest group of foreign-born residents with pulmonary tuberculosis in Japan in 2024 by country was from Nepal, up from fourth or fifth place between 2015 and 2023. In 2024, Nepal had 227 patients with pulmonary tuberculosis per 100,000 population. The number of Nepalese patients with pulmonary tuberculosis in Japan increased from 108 patients in 2015 to 293 patients in 2024. The relationship between the number of Nepalese residents with pulmonary tuberculosis and the Nepalese population in Japan is shown in Figure 4c.

#### 3.2.4. Relationship Between the Number of Burmese Patients with Pulmonary Tuberculosis and the Burmese Population in Japan

The fourth largest group of foreign-born residents with pulmonary tuberculosis in Japan in 2024 by country was from Myanmar, rising sharply from 23 patients in 2015 to 280 patients in 2024. In 2024, Myanmar had 428 patients with pulmonary tuberculosis per 100,000 population. The relationship between the number of Burmese residents with pulmonary tuberculosis and the Burmese population in Japan is shown in Figure 4d.

#### 3.2.5. Relationship Between the Number of Vietnamese Patients with Pulmonary Tuberculosis and the Vietnamese Population in Japan

The fifth largest group of foreign-born residents with pulmonary tuberculosis in Japan in 2024 by country was from Vietnam, increasing from 135 patients in 2015 to 331 patients in 2019 and averaging around 250 patients per year after 2020. The relationship between the number of Vietnamese residents with pulmonary tuberculosis and the Vietnamese population in Japan is shown in Figure 4e.

## 4. Discussion

In Japan, the number of patients with pulmonary tuberculosis had been steadily decreasing year by year, moving closer to realizing the public health goal of stamping out tuberculosis. However, the trend changed in 2022, and the total number of patients with pulmonary tuberculosis in Japan flatlined. This study revealed that foreign residents aged 20–39 years from countries with a higher incidence of pulmonary tuberculosis are at particularly high risk of developing the disease. The incidence of pulmonary tuberculosis in Japan may continue to increase as a result of the influx of immigrants, similar to the trends observed in Italy and Spain [9,10]. Therefore, the downsizing of tuberculosis units should be reconsidered. Tuberculosis units require isolated depressurized rooms and cannot be expanded easily. Tuberculosis units should only be reorganized after appropriate analysis.

In Japan, the technical intern training program was started in 1993, and factories and farms have accepted many foreign workers [11]. In addition, the Japanese government established a new status of residence in 2019, “specific skilled worker,” in order to supplement the shortage of Japanese workers due to population decline [12]. Accordingly, the population of foreign-born residents has increased, as shown in Figure 1b. The population of Japanese workers had been slowly declining before the COVID-19 pandemic as a consequence of Japan’s shrinking population. In addition to population decline, many older workers retired to avoid potential exposure to infection during the COVID-19 pandemic, and the number of workers in Japan decreased from 67.5 million in 2019 to 67.1 million in 2020 [13]. Thus, COVID-19 led to a severe shortage of workers. In contrast, the number of foreign workers increased from 1.66 million in 2019 to 2.30 million in 2024, raising the total number of workers in Japan to 67.8 million in 2024 [13,14,15]. Vietnamese workers accounted for the greatest number of foreign-born workers in Japan in 2024 at 0.57 million, followed by Chinese, Filipino, Nepalese, and Indonesian workers at 0.41, 0.25, 0.19 and 0.17 million, respectively. Burmese ranked seventh place at 0.11 million. The above seven countries have a high incidence of pulmonary tuberculosis, as shown in Table 2. Also, in terms of age distribution, 45.0% of foreign workers in the technical intern training program were aged 20–24 years and 82.4% were aged 20–39 years [16]. In other words, the number of workers aged 20–39 years has increased since 2022 as a result of the program, as shown in Figure 1b and Figure 3b, and the majority of them came from countries with a higher incidence of pulmonary tuberculosis, as shown in Table 2. This helps to explain the increase in the number of newly notified cases of pulmonary tuberculosis among foreign-born residents after 2022. In addition to the higher incidence rate, 2.8% (22/773) of foreign-born patients had multidrug-resistant *M. tuberculosis* (MDR-TB) in 2024, a far higher rate compared with the 0.6% rate (23/3741) for Japanese patients with MDR-TB among the 4514 newly notified pulmonary tuberculosis patients with known susceptibility test results [2,3]. The present findings suggest solutions to these problems.

Here, we discuss a possible reason why the decreasing trend of Japanese patients with pulmonary tuberculosis slowed after 2022. We think that the increased number of foreign-born patients influenced the incident of pulmonary tuberculosis among Japanese nationals because the lifestyles of foreign-born workers have changed. The majority of foreign workers came to Japan under the technical intern training program that started in 1993. Under this program, foreign workers studied in language schools and worked in factories or farms, living in dormitories where they formed small communities with other workers from their country of origin. These small communities were isolated from the larger Japanese society, and there were limited chances to interact with Japanese nationals. Therefore, when one of the workers developed pulmonary tuberculosis, the disease would spread in their small community, leading to epidemic outbreaks of pulmonary tuberculosis among foreigner workers [17,18]. Epidemic outbreaks of pulmonary tuberculosis in small communities are likely to occur in areas with many factories and farms but not in Tokyo, which has few factories and farms. The rate of pulmonary tuberculosis in a given prefecture will temporarily increase when there is an epidemic outbreak because the population there is relatively small [3]. However, it was rare for Japanese nationals to become infected. The COVID-19 pandemic ended after 2022, and more and more foreign workers have been recruited to fill the shortage caused by Japan’s shrinking population. In addition to the manufacturing and agriculture sectors, which were the targets of the technical intern training, the number of foreign-born workers in the retail, hospitality, health care, and service industries increased by 40.4%, 32.3%, 239.6%, and 33.0%, respectively, from 2019 to 2024 [14,15]. This may have contributed to the increased incidence of pulmonary tuberculosis among Japanese nationals after 2022. However, this is only one hypothesis, and transmission of tuberculosis from foreign-born residents to Japanese nationals cannot be established because there is no such evidence in the reports published by the Japanese government.

Next, we discuss the effects of the COVID-19 pandemic on the incidence of pulmonary tuberculosis. We expected that the incidence of pulmonary tuberculosis would be decreased by the COVID-19 pandemic because we thought the infection prevention measures for COVID-19 would also be effective against pulmonary tuberculosis. However, the number of pulmonary tuberculosis in Japan showed the same trend during the COVID-19 pandemic and did not greatly decrease, as shown in Figure 2 and Figure 3a. The COVID-19 pandemic did not affect pulmonary tuberculosis detection and notification, and the real reasons remain unclear.

As shown in Table 2, the top 5 countries of origin for foreign-born patients with pulmonary tuberculosis in Japan have a high incidence of the disease. Indonesian, Nepalese, and Burmese residents in Japan and their numbers of pulmonary tuberculosis notifications increased during the study period (Figure 4). In contrast, among Filipino and Vietnamese workers, a relationship between the numbers of residents with pulmonary tuberculosis and their populations in Japan was not observed. As one hypothesis, we consider that the risk of pulmonary tuberculosis in foreign-born residents differs according to their duration of stay in Japan. Foreign-born residents who have lived in Japan for a long period of time have a lower risk because the majority of permanent residents live in Japanese communities and Japanese nationals are at low risk of developing pulmonary tuberculosis. However, those who have recently come to Japan from countries with a higher incidence of pulmonary tuberculosis have a higher risk. The populations of Indonesian, Nepalese, and Burmese residents in Japan increased dramatically, rising from 35,910, 54,775, and 13,737 in 2015 to 199824, 233043 and 134574 in 2024, respectively [6]. The observed correlations indicate parallel temporal changes between the foreign-resident population and TB notifications for some countries; however, these associations cannot establish causality or transmission. The correlation between the numbers of Filipino residents with pulmonary tuberculosis and the Filipino population was not observed, because 62.6% of Filipinos in Japan were permanent residents with a low risk of pulmonary tuberculosis in 2024 [15]. In contrast, the Vietnamese population in Japan rose from 146,956 in 2015 to 411,968 in 2019, an increase of 180.3% [6]. A correlation between the numbers of residents with pulmonary tuberculosis and the Vietnamese population was observed between 2015 and 2019. The majority were new entrants who had a high risk of pulmonary tuberculosis. Subsequently, the Vietnamese population rose from 448,053 in 2020 to 634,361 in 2024, an increase of 41.6%. The number of Vietnamese long-term residents has increased since 2020, and this would be expected to affect the correlation, similar to the Filipino population.

It is expected that the population of Japanese nationals will continue to decline and the number of foreign-born workers will continue to increase. Accordingly, the number of patients with pulmonary tuberculosis in Japan may increase. To reduce the number of cases among foreign-born residents, the Japanese government has been conducting pre-entry tuberculosis screening of nationals from countries with a high incidence of cases who intend to come to Japan for work over the medium to long term to ensure that they do not have active tuberculosis before traveling to Japan [19,20]. The tuberculosis screening program has already been implemented in the Philippines, Nepal, and Vietnam, and is planned to be implemented in Indonesia, Myanmar, and China soon. A pre-entry tuberculosis screening program has been in operation in the United Kingdom since 2014 [21]. In addition, Australia, Canada, New Zealand, the United Kingdom, and the United States, who belong to the Immigration and Refugee Health Working Group, have similar pre-migration screening programs that are mandatory [22]. The number of patients with pulmonary tuberculosis in the United Kingdom decreased from around 11 per 100,000 population in 2014 to 7.8 patients per 100,000 population in 2022 before rising to 8.6 per 100,000 population in 2023. To understand the increased incidence in 2023, the number of tuberculosis screens was around 250,000 per year between 2014 and 2020; however, this increased after 2021, exceeding 1 million in 2023 due to the increasing number of migrants [21]. This suggests that screening by chest X-ray alone is not sufficient because tuberculosis has a long incubation period and chest X-rays cannot be used for diagnosis during the incubation period. Therefore, we recommend using the interferon gamma release assay, which has a higher diagnostic accuracy rate during the incubation period [23]. However, the high cost of the interferon gamma release assay may be prohibitive. As a next step, the Japanese government should implement pre-entry tuberculosis screening of Indonesian, Burmese, and Chinese workers as soon as possible. Then, the Japanese government will need to regularly evaluate the effects of the screening program and make improvements where possible. The effects of the screening program are not clear at present, and thus the reorganization and downsizing of tuberculosis units should be reconsidered for the time being.

A major limitation of this study is that it involved an ecological analysis of aggregate surveillance data, and the population and tuberculosis data were not obtained from the same sources, and the methods of collection differed according to the ministry and agency. In addition, some results cannot be supported with evidence because detailed information such as the mode of infection, date of entry into Japan, onset date, diagnosis date, severity, and so on were not published in the reports by the Japanese government. Therefore, the incidence of pulmonary tuberculosis according to the number of foreign workers entering Japan could not evaluated.

## 5. Conclusions

Pulmonary tuberculosis in Japan had been decreasing as a result of the country’s anti-tuberculosis policy. However, the decreasing incidence trend ended in 2022. This study revealed that foreign residents aged 20–39 years from countries with a higher incidence of pulmonary tuberculosis are at particularly high risk. The population of Japanese nationals continues to decline and the number of foreign-born workers in Japan has increased. The changing composition of the population has impacted the incidence of pulmonary tuberculosis in Japan, making it difficult to reduce the number of future cases in Japan. To decrease number of patients with tuberculosis in Japan, more effective pre-entry tuberculosis screening should be introduced for young migrant workers from countries with a high incidence of tuberculosis cases.

## Figures and Tables

**Table 1:** Age-standardized rates of newly notified cases of pulmonary tuberculosis per 100,000 population among Japanese nationals and foreign-born residents in Japan.

	2015	2016	2017	2018	2019	2020	2021	2022	2023	2024
Japanese nationals	13.8	13.0	12.1	11.3	10.4	9.2	8.1	7.2	6.8	6.6
Foreign-born residents	66.9	49.9	53.3	53.2	47.7	41.6	35.7	31.4	39.4	37.4

**Table 2:** Numbers of newly notified cases of pulmonary tuberculosis among foreign-born patients in Japan for each country of origin and the rates of newly notified cases of pulmonary tuberculosis in these countries 2024.

Country	Number of Patients from Country in Japan	Newly Notified Cases in Country ^1^
Indonesia	423	382
Philippine	331	625
Nepal	293	227
Myanmar	280	482
Vietnam	252	182
China	104	49
India	40	187
Thailand	26	146
Cambodia	23	272
Republic of Korea	21	35

^1^ Number of patients with pulmonary tuberculosis per 100,000 population.

## Data Availability

This study analyzed publicly available data.

## Abbreviations

The following abbreviations are used in this manuscript:

COVID-19	Coronavirus Disease 2019
MDR-TB	Multidrug-resistant *M. tuberculosis*
